# Efficacy of ketogenic diet in CDKL5-related epilepsy: a single arm meta-analysis

**DOI:** 10.1186/s13023-022-02492-6

**Published:** 2022-10-23

**Authors:** Jie Zhang, Jiayi Ma, Xuting Chang, Pengxia Wu, Shangru Li, Ye Wu

**Affiliations:** Department of Pediatrics, Peking University First Hospital, No. 1 Xi’an Men Street, West District, Beijing, 100034 China

**Keywords:** Ketogenic diet, Epilepsy, CDKL5, Meta-analysis

## Abstract

**Background:**

Drug-resistant epilepsy is one of the most important features of cyclin-dependent kinase-like 5 (CDKL5) deficiency disorder. The ketogenic diet (KD) may be effective for patients with CDKL5-related epilepsy, but there is little high-quality evidence to confirm the efficacy. This meta-analysis investigated the efficacy and safety of KD in CDKL5-related epilepsy.

**Methods:**

The PubMed, Embase, Web of Science, Cochrane Library, WanFang, CNKI and VIP databases were searched for relevant studies published up to January 1, 2022. Two reviewers independently screened the literature according to inclusion and exclusion criteria and evaluated the bias risk of the included studies. Meta-analysis was performed using Review Manager 5.3 software.

**Results:**

A total of 12 retrospective studies involving 193 patients met the inclusion criteria. Meta-analysis revealed that the definite responder rate to KD in the treatment of CDKL5-related epilepsy was 18.0% [95% CI (0.07, 0.67)], with no statistical heterogeneity among studies (I^2^ = 0%, *P* = 0.45). The clinical responder rate was 50.5% [95% CI (0.75, 1.39)], and there was no statistical heterogeneity among all studies (I^2^ = 46%, *P* = 0.05). Subgroup analysis showed that there was no significant difference in the clinical responder rate between the two groups with seizure onset age before and after 1 month (*P* = 0.14). Only one study mentioned adverse reactions, and the incidence of adverse reactions was 78.3% (18/23). Constipation and vomiting were the main manifestations, implying a high incidence of gastrointestinal adverse reactions.

**Conclusions:**

The definite responder rate to KD in CDKL5-related epilepsy was 18%, and the gastrointestinal adverse reactions were probably common in these patients. All the studies included in the meta-analysis were retrospective, and most of them had small sample sizes. Additional high-quality studies are needed to confirm the efficacy and tolerance of KD in CDKL5-related epilepsy.

## Background

*CDKL5* (cyclin-dependent kinase-like 5) is located in the short arm of X chromosome (Xp22) [1]. The protein encoded by *CDKL5* is a serine/threonine kinase that is widely expressed in human tissues, predominantly in the brain [2]. The CDKL5 protein plays an important role in cell proliferation, neuronal migration, axonal outgrowth, dendritic morphogenesis and synapse development [3–5]. CDKL5 deficiency disorder (CDD, OMIM 300672), also known as CDKL5 encephalopathy, CDKL5 epileptic encephalopathy, or CDKL5-related epilepsy, is a rare neurological disorder. Pathogenic variants in CDKL5 cause early-life epilepsy in 1 per 40,000–60,000 live births [6, 7]. CDKL5 deficiency disorder is linked to the X chromosome and affects the female gender four times more often than men, which suggests that it is mostly a lethal mutation in male fetal life. Clinical features include infantile-onset refractory epilepsy, developmental delay and intellectual disability. Most of the patients are unable to walk or speak independently. Sleep disorders, gastroesophageal reflux, visual impairment and other multisystem damage are also common manifestations of CDD [3, 8–14]. Seizure is one of the most important manifestations of CDD, with a median onset age of 5 weeks after birth (range from 3 to 8 weeks). Spasms, tonic, and tonic–clonic seizures are the most common types of seizures [15], with a poor response to most antiseizure drugs. Refractory epilepsy severely impacts quality of life and neurodevelopment.

Ketogenic diet (KD) has been proven to be effective for drug-resistant epilepsy and a variety of seizure types [16]. It also tends to be associated with improved neurobehavioral development of children with drug-resistant epilepsy [17]. Updated recommendations of the International Ketogenic Diet Study Group in 2018 suggested that KD was moderately beneficial for CDKL5-related epilepsy [18]. However, the quality of the evidence was poor because the data came from a retrospective observational study with a relatively large sample size [19], and the efficacy of KD in CDKL5-related epilepsy was not clear. CDKL5-related epilepsy is a rare nervous system disease, and high-quality evidence from studies with large samples, prospective cohorts or randomized control trials is relatively difficult to achieve. The efficacy of KD in CDKL5-related epilepsy is still not clear, so we performed a meta-analysis based on previous studies to collect evidence regarding the efficacy of KD and to provide a basis for the treatment of these patients.

## Methods

### Search strategy

**Fig. 1:**
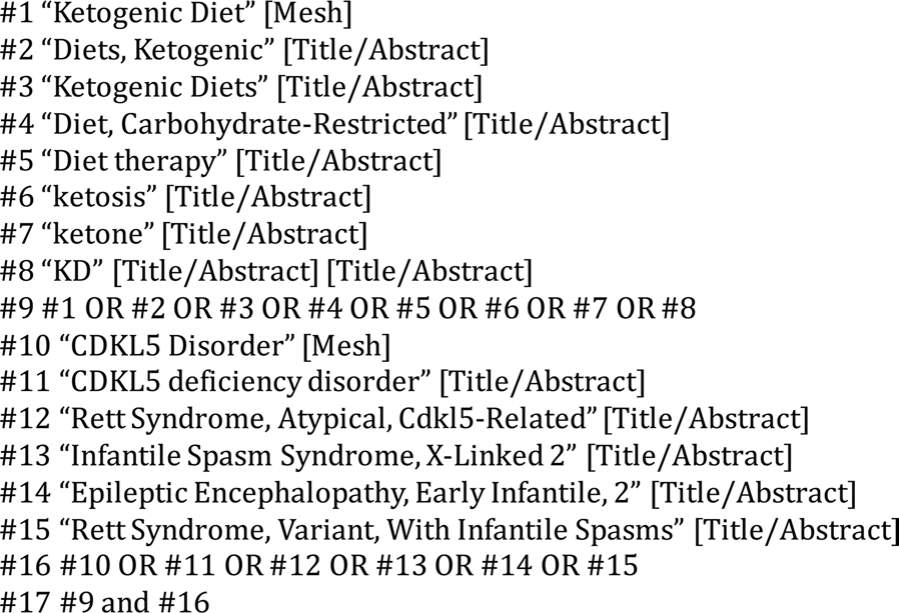
Searching strategy of literature

This systematic review was conducted according to the Preferred Reporting Items for Systematic Reviews and Meta-Analyses (PRISMA) guidelines [20]. We identified relevant studies using the following medical subject headings (MeSH) terms: "Ketogenic Diet [Mesh]”, “CDKL5 Disorder [Mesh]”, and key words such as “Diets, Ketogenic”, “Ketogenic Diets”, “Diet, Carbohydrate-Restricted”, “Diet therapy”, “ketosis”, “ketone”, “KD”, “CDKL5 deficiency disorder”, “Rett Syndrome, Atypical, Cdkl5-Related”, “Infantile Spasm Syndrome, X-Linked 2”, “Epileptic Encephalopathy, Early Infantile, 2″, and “Rett Syndrome, Variant, With Infantile Spasms” (Fig. 1). The PubMed, Embase, Web of Science, Cochrane Library, WanFang, CNKI and VIP databases were searched. The final search was carried out January 1, 2022.

### Inclusion and exclusion criteria

Randomized controlled trials (RCTs), nonrandomized controlled trials, case–control studies, cohort studies, and case series were included. Studies published in English and Chinese were included. The inclusion criteria were as follows: (1) research types: randomized controlled trials (RCTs), nonrandomized controlled trials, case–control studies, cohort studies, and case series were included; (2) research objects: patients with epilepsy who had *CDKL5* mutations; (3) interventions: patients received KD therapy; there were no restrictions on whether a control group was set.

Articles were excluded if they met the following criteria: (1) reviews, animal or in vitro studies; (2) no clear outcomes; and (3) literature not in English or Chinese.

### Outcomes

#### Primary outcomes

*Definite Responder Rate:* The proportion of patients with seizure frequency reduced ≥ 50% from baseline after KD therapy.

#### Secondary outcomes

1. *Clinical Responder Rate*: The parents or doctors judged the KD therapy to be “clinically effective” by reducing the seizure frequency by at least 50% or the percentage of reduction was unknown. The proportion of these patients was the clinical responder rate;
2. *Clinical seizure-free rate*: The proportion of seizure-free patients at the last follow-up after KD therapy;
3. *Adverse reaction rate*: The incidence of adverse reactions during KD therapy, including vomiting, diarrhea, feeding difficulty, lethargy, significant weight loss (≥ 10%), acidosis, urinary calculi and so on.

### Literature screening, data extraction, and methodological quality assessment

Each study was independently assessed by two review authors (ZJ and MJY) to extract the relevant information, and the filtered information was crosschecked to ensure the integrity, objectivity, and correctness of the data. A third author (WY) checked all articles again to ensure correct interpretation of the data. Discrepancies were resolved through discussion and consensus. For all included articles, the following data were extracted when available: (1) information of the studies: title, first author, year of publication, type of study; (2) baseline data of the subjects: adults or children, sample size, gender, age of seizure onset, type of seizure, frequency of seizure; (3) details of KD: age of initiation, treatment duration, reduction of seizure frequency, time of follow-up, adverse reactions.

Two review authors independently assessed the risk bias of the included studies. The methodological quality of randomized controlled trials was reviewed using the Cochrane risk of bias tool, and the observational studies were reviewed using the Newcastle–Ottawa scale (NOS) [21].

### Statistical analysis

The analysis for outcomes was descriptive, and data were summarized as the median and mean. RevMan software (version 5.3) was utilized for the statistical analysis. The heterogeneity of studies was evaluated with the I^2^ test. If there was no statistical heterogeneity among the studies (I^2^ ≤ 50%), the fixed effects model was used for meta-analysis. If there was statistical heterogeneity (I^2^ > 50%) among the results, the source of heterogeneity was further analyzed. After excluding the influence of obvious clinical heterogeneity, the random effects model was used for meta-analysis. Significant clinical heterogeneity was dealed with subgroup analysis, sensitivity analysis, or descriptive analysis only. The test level of the meta-analysis was set as α = 0.05. Subgroup analysis was performed according to the onset age before and after 1 month to compare the differences in efficacy. A funnel plot was used to test whether publication bias existed.

## Results

### Search results

**Fig. 2:**
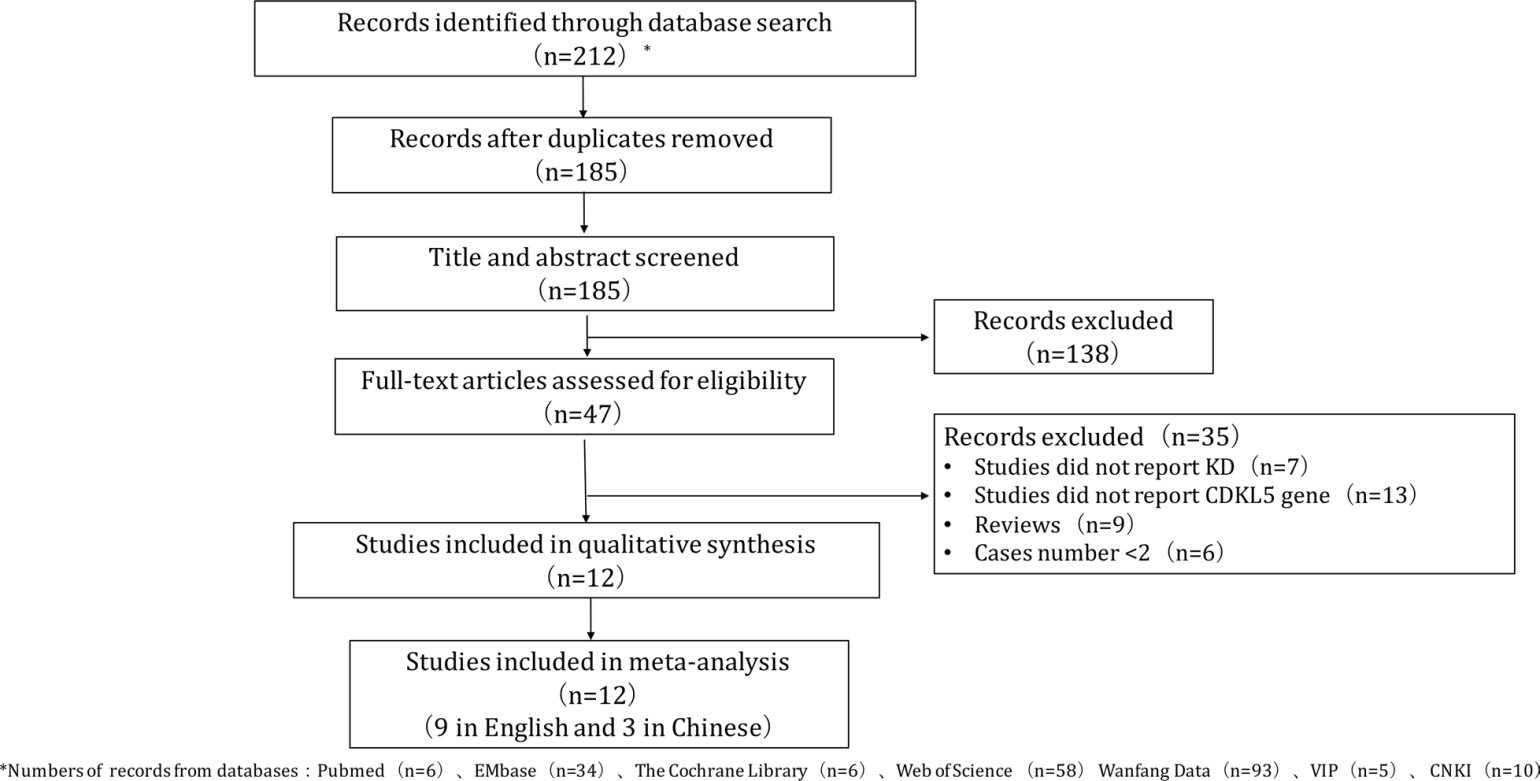
Flow chart of literature screening

Figure 2 detailed the search results. A total of 212 potentially relevant publications were systematically identified (104 records in English databases and 108 records in Chinese database). 27 duplicate records were removed. 138 studies were removed after the two independent reviewers read the titles and abstracts. Thirty-five studies were excluded after reading the full texts. Ultimately, 12 studies (9 studies in English and 3 studies in Chinese) with 193 patients were included for literature analysis [8, 19, 22–31].

### Basic characteristics and bias in the included studies

**Fig. 3:**
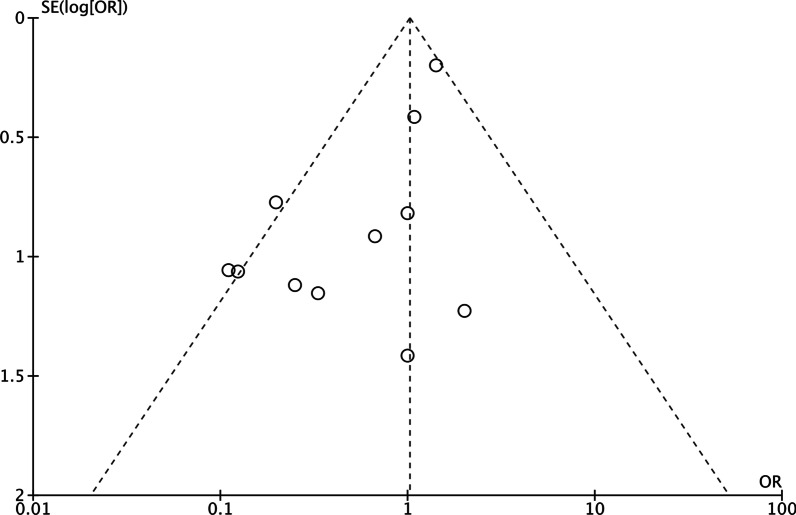
Funnel plot of the responder rate

The basic characteristics of the included studies were shown in Table 1. As no RCT was included in our analysis, the risk of bias assessment was performed using the Newcastle–Ottawa scale (NOS). The NOS results for the 12 studies were 3–5, as shown in Table 1. The funnel plot of the responder rate to KD in CDKL5-related epilepsy was shown in Fig. 3. The results showed that the funnel plot was asymmetric, which might indicate publication bias due to the small sample sizes of the included studies, lack of statistical significance, and some negative results not being published.

**Table 1:** Basic data and bias in the included studies

Included studies	Type of studies	Cases	Median age of KD initiation	Intervention		Time of Follow-up	Outcomes		Responder rate%	NOS
	(Cohort)			T	C	(Median)			(n/N)	
Amin [23]	Retrospective	23	22 months	KD	–	2 years	Only described effective (parent questionnaire)	52.2(12/23)		5
Ko [26]	Retrospective	9	Not mentioned	KD	–	Not mentioned	Only described effective	11.1(1/9)		3
Ko [25]	Retrospective	10	Not mentioned	KD	–	≥ 12 months	Seizure reduction ≥ 90%	0(0/10)		5
Kobayashi [27]	Retrospective	10	Not mentioned	KD	–	Not mentioned	Seizure reduction ≥ 50%	10.0(1/10)		4
Lim [19]	Retrospective	104	Not mentioned	KD	–	17 months	Only described effective (parent questionnaire)	58.7(61/104)		5
Moseley [8]	Retrospective	6	Not mentioned	KD	–	Not mentioned	Only described effective (parent questionnaire)	50.0(3/6)		4
Müller [22]	Retrospective	12	Not mentioned	KD	–	≥ 3 months	Seizure reduction ≥ 50% in last 4 weeks	16.7(2/12)		5
Neupauerová [24]	Retrospective	2	3 years	KD	–	Not mentioned	Seizure reduction ≥ 50%	50.0(1/2)		4
Siri [28]	Retrospective	5	Not mentioned	KD	–	Not mentioned	Only described effective	20.0(1/5)		3
Hu [29]	Retrospective	4	Not mentioned	KD	–	Not mentioned	Only described effective	25.0(1/4)		3
Xiong [30]	Retrospective	5	Not mentioned	KD	–	Not mentioned	Only described effective	40.0(2/5)		3
Mei [31]	Retrospective	3	Not mentioned	KD	–	Not mentioned	Only described effective	66.7%(2/3)		3

KD = ketogenic diet; T = trial group; C = control group; NOS = Newcastle–Ottawa scale

### Systematic review (qualitative analysis)

A total of 12 retrospective studies were included in the qualitative analysis [8, 19, 22–31]. Among all the studies, 193 patients were treated with KD. All the studies mentioned the efficacy of KD in CDKL5-related epilepsy, and four of them [22, 24, 25, 27] clearly defined the efficacy, with an overall responder rate of 11.8% (4/34). One study [22] clearly defined the efficacy as seizure reduction ≥ 50% in the last 4 weeks. A total of 12 patients were included in this study, with a follow-up time of more than 3 months, and 2 patients were definitely responders, with a definite responder rate of 16.7% (2/12). Two studies [24, 27] defined the efficacy as seizure reduction ≥ 50%. A total of 12 patients were included, with no follow-up time mentioned, and the definite responder rate was 16.7% (2/12). One study [25] defined the efficacy as seizure reduction ≥ 90%, and a total of 10 patients were included. All patients were followed up for at least 12 months, and the responder rate was 0%. The remaining 8 studies [8, 19, 23, 26, 28–31] did not clearly define the efficacy, and a total of 159 patients were included. The responder rate was 52.2% (83/159). Among them, 2 studies [19, 23] mentioned that the median time of follow-up after KD was 2 years and 17 months, and 2 studies [22, 25] mentioned that the median time of follow-up was at least 3 months and 12 months. Follow-up time was not mentioned in other studies. Among them, 3 studies [19, 22, 23] did not define the efficacy, and the efficacy was only assessed by the parents through parent questionnaires. The remaining 5 studies [26, 28–31] only described the treatment as effective but did not define the efficacy. Five studies [22, 24, 27, 29, 30] mentioned seizure-free patients after KD. A total of 27 patients were included, 2 of whom were seizure free, and the overall rate of seizures was 7.4%. One patient achieved seizure-free status after 3 months of KD. The other case did not mention when to achieve seizure free and was seizure free for 6 months, and abnormal discharge disappeared in the electroencephalogram at the last follow-up.

Regarding safety, only one study [23] mentioned adverse reactions during KD. A total of 23 patients were included in this study, and 18 patients showed adverse reactions, such as constipation and vomiting.

### Results of the meta-analysis

#### Primary outcome

##### Definite responder rate

**Fig. 4:**
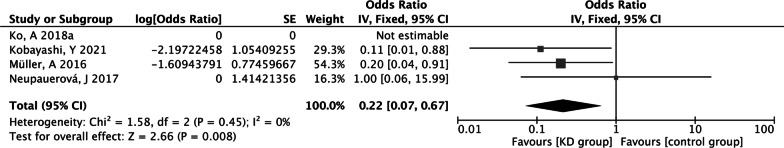
Forest plot of definite responder rate

Four studies [22, 24, 25, 27] including a total of 34 patients clearly defined the efficacy as seizure reduction ≥ 50% after KD. Meta-analysis was conducted using the fixed effects model and showed that the ORR of the definite responder rate was 0.22 [95% CI (0.07, 0.67)], and the definite responder rate after conversion by ORR was 18.0% [ORR/(1 + ORR)]. There was no statistical heterogeneity among the studies (I^2^ = 0%, *P* = 0.45) (Fig. 4).

#### Secondary outcomes

##### Clinical responder rate

**Fig. 5:**
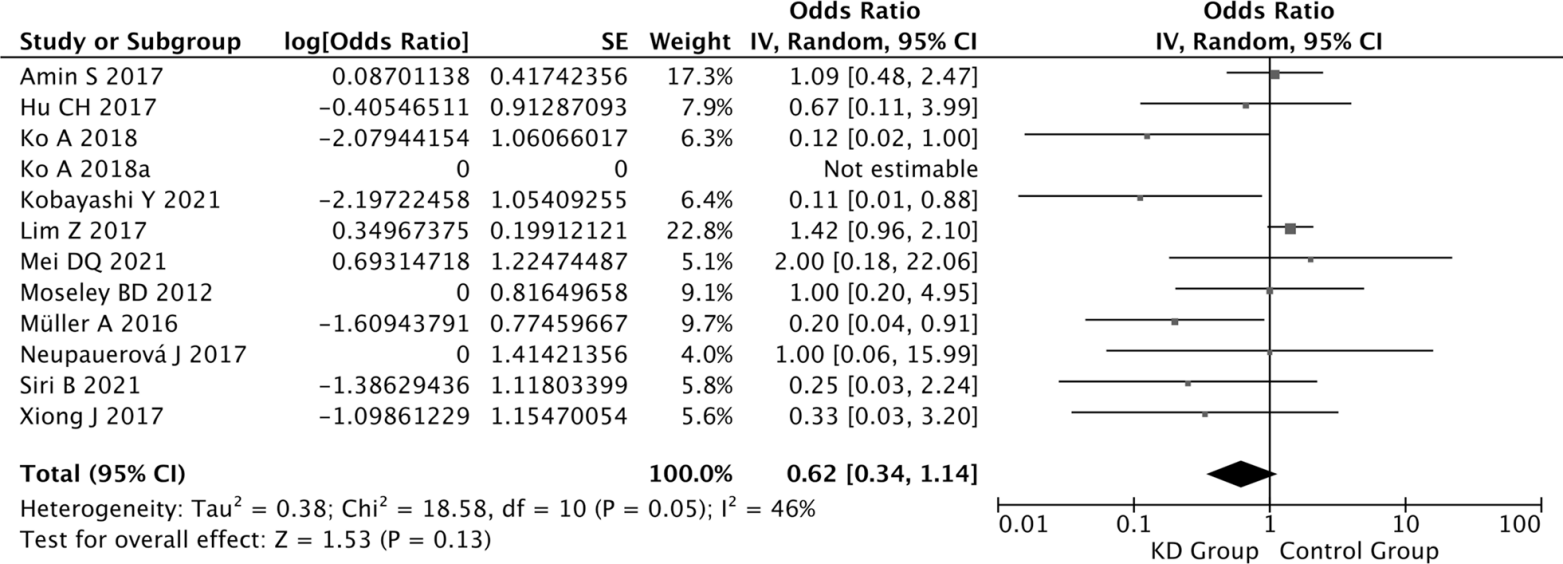
Forest plot of clinical responder rate

The parents or doctors judged that KD therapy was clinically effective by reducing the seizure frequency by at least 50%, or the percentage of reduction was unknown. Twelve studies [8, 19, 22–31] including a total of 193 patients reported this outcome. Meta-analysis was conducted using the fixed effects model and showed that the ORR of the clinical responder rate was 1.02 [95% CI (0.75, 1.39)], and the clinical responder rate after conversion by ORR was 50.5% [ORR/(1 + ORR)]. There was no statistical heterogeneity among the studies (I^2^ = 46%, *P* = 0.05) (Fig. 5).

##### Clinical seizure-free rate

Five studies [22, 24, 27, 29, 30] mentioned seizure-free patients after KD therapy, but they did not provide details about the seizure-free duration and follow-up time. Meta-analysis was not conducted to pool the results for the seizure-free rate because the sample sizes in the five studies were all small (≤ 10 cases), including 10 cases, 6 cases, 2 cases, 4 cases and 5 cases. A total of 27 patients were included, of which 2 achieved seizure-free status, with a seizure-free rate of 7.4%. One patient achieved seizure-free status after 3 months of KD. The other case did not mention when to achieve seizure free and was seizure free for 6 months, and abnormal discharge disappeared in the electroencephalogram at the last follow-up.

##### Subgroup analysis

**Fig. 6:**
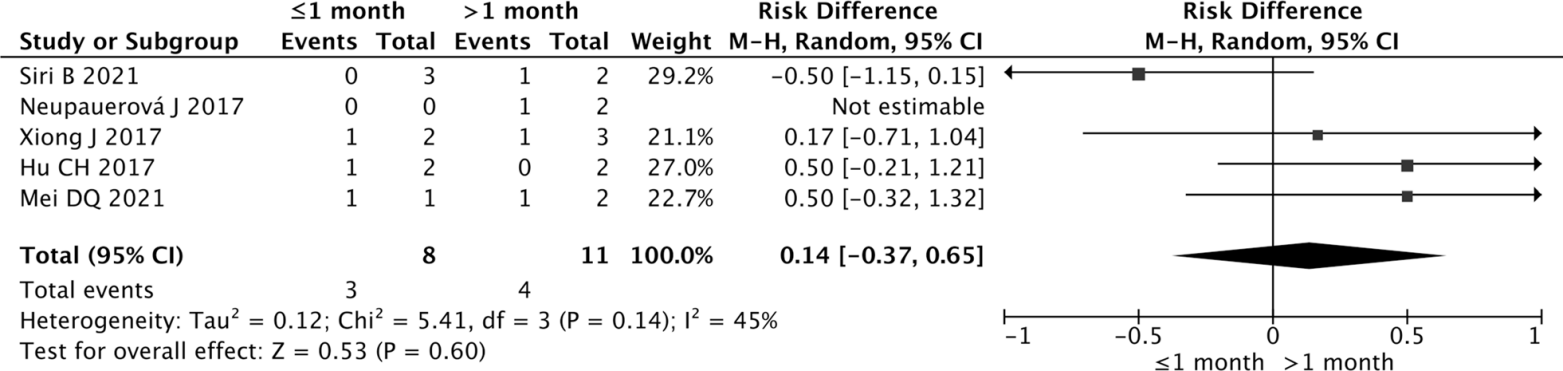
Forest plot of clinical responder rate in subgroup analysis (onset age before or after 1 month)

Patients were divided into two groups based on the onset age of seizure: onset age before or after 1 month. A total of 19 patients were included in 5 studies [24, 28–31], including 8 patients with onset age of seizure before 1 month and 11 children with onset age after 1 month. Meta-analysis was conducted using the fixed effects model and showed that there was no significant difference in the clinical responder rate between the two groups (*P* = 0.14) (Fig. 6).

## Discussion

CDKL5 deficiency disorder is a rare neurological disorder leading to high morbidity and mortality. Epileptic encephalopathy is one of the most important manifestations and often shows a poor response to a variety of anti-seizure medications. There are no specific effective medications for this disorder, and there is no definite evidence for the efficacy of KD. As CDKL5 deficiency disorder is extremely rare, high-quality evidence from large-sample, randomized or prospective studies is relatively difficult to achieve. We performed a meta-analysis on KD in patients with CDKL5-related epilepsy to provide relatively reliable evidence and a data basis for future research.

### The definite responder rate to KD in CDKL5-related epilepsy was low

The purpose of this meta-analysis was to determine the efficacy of KD in CDKL5-related epilepsy and to evaluate its possible adverse reactions. Twelve retrospective studies were included in this analysis. The responder rate varied widely from 0 to 66.7% among different studies. Among them, 4 studies [22, 24, 25, 27] clearly defined the efficacy of KD therapy (seizure reduction ≥ 50%), and the responder rates were 0% (0/10), 10.0% (1/10), 16.7% (2/12), and 50.0% (1/2). For the results of the meta-analysis, the definite responder rate was only 18.0%. The remaining 8 studies [8, 19, 23, 26, 28–31] did not clearly define the efficacy, and the responder rates reported in each study ranged from 11.1% to 66.7%. For the results of the meta-analysis including all twelve studies, the clinical responder rate was 50.5%, which was much higher than the defined responder rate. The difference resulted from the fact that most of the studies did not define the responder as an outcome of efficacy. The outcomes were relatively unclear and could not represent the real efficacy of KD, which might overestimate the efficacy to some degree. A meta-analysis with 5 randomized controlled trials involving 472 children confirmed that the definite responder rate (seizure frequency reduction ≥ 50%) to KD in the treatment of children with drug-resistant epilepsy was 52.0% [32]. Compared with the efficacy of KD in drug-resistant epilepsy [32], the definite responder rate to KD in CDKL5-related epilepsy was low (18.% vs. 52.0%), and the literature with low-quality evidence might overestimate the efficacy in our study. More high-quality research evidence is needed in the future to confirm the efficacy.

### The incidence of gastrointestinal adverse reactions was high, and these were the most common adverse reactions.

For the safety of KD, a meta-analysis with five randomized controlled trials involving 472 children with refractory epilepsy [32] showed that gastrointestinal adverse reactions were the most common adverse reactions, with an incidence of 30%, manifested as vomiting, diarrhea and constipation. In this analysis, only one study [23] mentioned adverse reactions during the KD, and the incidence of adverse reactions was 78.3% (18/23), manifested as constipation and vomiting. The incidence of gastrointestinal adverse reactions was relatively higher in the patients with CDKL5-related epilepsy during the KD. On the one hand, this might be related to the bias of low-quality evidence from the studies with small samples. On the other hand, CDKL5 deficiency disorder itself is prone to gastroesophageal reflux [8], while KD is a specific diet with a high fat ratio, which might be more likely to cause gastrointestinal adverse reactions. Further studies with higher quality are needed to confirm the safety of KD in CDKL5-related epilepsy.

### Retrospective studies with low quality of evidence and incomplete details about KD might affect the reliability of the final results.

All 12 studies included in the analysis were retrospective studies, and the quality of evidence of the studies was relatively low. Few studies have specifically targeted the efficacy of KD in CDKL5-related epilepsy. In this analysis, only two studies [19, 22] were targeted to analyze the efficacy of KD in CDKL5-related epilepsy. The main purpose of most studies [8, 23–25, 27–31] was to analyze the phenotypes related to *CDKL5* gene mutations, and the efficacy was mentioned in the article, but without the specific details of KD therapy. Four studies [19, 22, 23, 25] clearly mentioned the follow-up time after KD, and the median time of follow-up in two studies was 2 years and 17 months, respectively. The other two studies performed at least 3 months and 12 months of follow-up. The follow-up time was reasonable in these four studies, and the efficacy was relatively reliable. The other 8 studies did not mention the follow-up time after KD, which might lead to an inaccurate evaluation of the real efficacy.

Regarding the outcomes to evaluate the efficacy of KD, the responders were clearly defined as having a seizure frequency reduced by more than 50% after KD only in only 4 studies [22, 24, 25, 27]. The responders in the remaining 8 studies did not clearly define the outcomes of efficacy. Among the 8 studies, the efficacy was evaluated through parent questionnaires without clear definition in 3 studies [19, 22, 23]. The outcomes were only described as “effective” in the articles without specific descriptions of seizure frequency reduction in the other 5 studies [26, 28–31]. In general, outcomes of efficacy were inaccurate in most studies, and the lack of detailed descriptions of KD might affect the final evaluation of the real efficacy of KD.

In addition, the sample sizes of the included studies varied greatly, ranging from 2 to 104 cases. The sample sizes in 9 studies [19, 22, 23] were less than 10 cases. The small sample sizes might affect the accuracy of the meta-analysis results. Lim Z [19] performed the study with the largest sample size among the included studies, and the clinical responder rate was 58.7% (61/104). The relatively high responder rate might be related to the unclear definition of the efficacy and retrospective questionnaire for parents, which might lead to recall bias and selection bias.

### Limitations

There were some limitations in this study. (1) All the included studies for meta-analysis were retrospective studies with low quality, and there was considerable heterogeneity among the studies. The accuracy of the results may be affected by the lack of multicenter, large-sample studies and small sample sizes in most included studies. (2) The efficacy was not strictly defined in most studies, and the outcomes were only described as “effective”, which was only judged by the parents or doctors, without specific descriptions of seizure frequency reduction. The relatively subjective judgments without a clear definition of the efficacy might affect the accuracy of the results. (3) A detailed description of KD therapy was not available in some studies. There might be some clinical heterogeneity among different studies caused by epileptic syndromes, seizure types, or whether anti-seizure drugs were adjusted during KD. Additional studies with larger sample sizes, higher quality, and more homogeneity are needed to confirm the efficacy of KD.

## Conclusions

The systematic review and meta-analysis showed that the definite responder rate of KD in CDKL5-related epilepsy was only 18.0%. The incidence of gastrointestinal adverse reactions was high in some studies, so close attention should be devoted to gastrointestinal reactions during KD therapy. However, the included studies were all retrospective studies with low quality, and the sample sizes of most studies were small. Further prospective studies with large samples are needed to confirm the efficacy of KD in CDKL5-related epilepsy.

## Data Availability

All data generated or analyzed during this study are included in this published article and its additional information files.
